# Cysteine redox state regulates human β2-adrenergic receptor binding and function

**DOI:** 10.1038/s41598-020-59983-4

**Published:** 2020-02-19

**Authors:** Kalyn M. Rambacher, Nader H. Moniri

**Affiliations:** Department of Pharmaceutical Sciences, College of Pharmacy, Mercer University Health Sciences Center, Mercer University, Atlanta, GA30341 United States

**Keywords:** Hormone receptors, Respiration

## Abstract

Bronchoconstrictive airway disorders such as asthma are characterized by inflammation and increases in reactive oxygen species (ROS), which produce a highly oxidative environment. β2-adrenergic receptor (β2AR) agonists are a mainstay of clinical therapy for asthma and provide bronchorelaxation upon inhalation. We have previously shown that β2AR agonism generates intracellular ROS, an effect that is required for receptor function, and which post-translationally oxidizes β2AR cysteine thiols to Cys-S-sulfenic acids (Cys-S-OH). Furthermore, highly oxidative environments can irreversibly oxidize Cys-S-OH to Cys-S-sulfinic (Cys-SO_2_H) or S-sulfonic (Cys-SO_3_H) acids, which are incapable of further participating in homeostatic redox reactions (i.e., redox-deficient). The aim of this study was to examine the vitality of β2AR-ROS interplay and the resultant functional consequences of β2AR Cys-redox in the receptors native, oxidized, and redox-deficient states. Here, we show for the first time that β2AR can be oxidized to Cys-S-OH *in situ*, moreover, using both clonal cells and a human airway epithelial cell line endogenously expressing β2AR, we show that receptor redox state profoundly influences β2AR orthosteric ligand binding and downstream function. Specifically, homeostatic β2AR redox states are vital toward agonist-induced cAMP formation and subsequent CREB and G-protein-dependent ERK1/2 phosphorylation, in addition to β-arrestin-2 recruitment and downstream arrestin-dependent ERK1/2 phosphorylation and internalization. On the contrary, redox-deficient β2AR states exhibit decreased ability to signal via either Gαs or β-arrestin. Together, our results demonstrate a β2AR-ROS redox axis, which if disturbed, interferes with proper receptor function.

## Introduction

Inhaled β2-adrenergic receptor (β2AR) agonists are efficacious bronchodilators that are used clinically in patients with pulmonary disorders such as asthma or chronic obstructive pulmonary disease (COPD), the hypercontractility of which is linked to pulmonary inflammation and a highly oxidative environment. In these disease states, the pulmonary epithelia and underlying smooth muscle contain elevated concentrations of reactive oxygen species (ROS), and the membrane-bound ROS generating enzyme NADPH oxidase (NOX) is known to be upregulated^1–3^. Of note, NOX4 is overexpressed in the lungs of asthmatic patients compared to healthy controls, while NOX2, NOX4 and the related DUOX2 oxidases are upregulated in airway epithelia of COPD patients, increasing the total burden of oxidative stress in both diseases^2,4^. Moreover, agonism of β2AR activates NOX isoforms and induces ROS generation, an effect that has been shown to occur in a myriad of cell lines and tissues including cardiomyocytes^5–7^, neuroglial cells^8^, aortic tissue^9^, and the COS-7^6^ and HEK293^10–12^ clonal cell lines, suggesting that clinical treatment with inhaled β2AR agonists may contribute to the oxidizing pulmonary environment. Given the importance of β2ARs in the treatment of airway diseases and despite overwhelming evidence that β2-agonists are utilized in the presence of heightened ROS in the lung, most pharmacological data on β2AR function is derived in non-oxidative environments, which fail to address the impact ROS may exert on β2AR function. Indeed, it is known that chronic use of β2-agonists decreases their clinical efficacy, leading to tachyphylaxis of the bronchodilating effects^13^.

Intracellular ROS (e.g. H_2_O_2_) can both transiently and/or irreversibly oxidize cysteine residues depending upon the oxidizing potential and concentration of ROS, and/or the duration of ROS exposure. Transient first order cysteine oxidation, known as cysteine S-sulfenation (Cys-S-OH)^14^, has recently emerged as a form of post-translation modification that exerts significant effects on protein function, similar to that of phosphorylation. For example, GAPDH requires Cys149 to be S-sulfenated for enzymatic function^15,16^, S-sulfenation of Cys797 of the epidermal growth factor receptor (EGFR) enhances RTK activity^17^, and S-sulfenation of β/γ-actin is known to inhibit polymerization^18^. Conversely, higher order oxidation to irreversible S-sulfinic (Cys-SO_2_H) and S-sulfonic (Cys-SO_3_H) acids upon prolonged ROS exposure or in highly oxidative environments prevent involved residues from normal reduction and inhibits further transient redox chemistry^14^, making such residues redox-deficient and incapable of conventional redox cycling. There is evidence that cysteine redox state modulates β2AR function, as thiol alkylation locks the receptor in a G-protein bound complex^19^, and further, the presence of redox modulators alters β2AR function and ligand binding through a mechanism we now know to be cysteine S-sulfenation^20^. Importantly, our laboratory first revealed that the β2AR protein is in fact S-sulfenated following agonism with the fully efficacious agonist isoproterenol (ISO), an effect that is blocked by the β-antagonist propranolol and mimicked by exogenous H_2_O_2_ treatment^21^. Furthermore, we have previously shown that both inhibition of ROS generation or ROS scavenging significantly decreases activation of both G-protein-dependent and β-arrestin mediated β2AR signaling pathways^10,11^, demonstrating that ROS-mediated S-sulfenation exerts functional consequences upon β2AR. Therefore, in an oxidative milieu, ROS may be modifying receptor cysteine residues that then exert functionally distinct agonist-mediated signaling profiles than seen with the non-oxidized receptor counterparts.

The goal of the current study was to determine the role of oxidant-mediated cysteine S-sulfenation of the β2AR on receptor function. To better elucidate the functional role of β2AR cysteine-S-sulfenation and their physiological implications, we utilized three receptor models, representing: 1) the “native” state of the receptor that is capable of normal redox cycling, 2) an “oxidized” state of the receptor that is known to be Cys-S-sulfenated in the presence of H_2_O_2_^21^ and 3) a “redox-deficient” state that irreversibly blocks transient redox activity.

## Materials and Methods

### Reagents and chemicals

DYn-2 (250 mM in DMSO) was from Kerafast (Boston, MA). CM-H2DCFDA (10 µM prepared fresh in DMSO) was from Thermo Fisher (Waltham, MA). Biotin-azide (5 mM in DMSO) was from Cayman Chemical (Ann Arbor, MI). FITC conjugated anti-mouse antibody was from Rockland Immunochemicals (Limerick, PA). [^3^H]-Dihydroalprenolol was purchased from Perkin Elmer (Waltham, MA). All other reagents were purchased from Sigma-Aldrich or VWR at their highest available purities.

### Constructs

The FLAG-epitope tagged pcDNA3-FLAG-β2AR construct was a kind gift from Dr. Robert J. Lefkowitz (Duke University Medical Center, Durham, NC). The Cysteine 341 mutant was made by polymerase chain reaction using the Quick-Change II XL sit-directed mutagenesis kit (Agilent Technologies, Cedar Creek, TX). The dynamin K44A plasmid was a kind gift from Dr. Yehia Daaka (University of Florida, Gainesville, FL). The P(EGF-topaz)-rat-β-arrestin-2-YFP plasmid was a kind gift from Dr. Michel Bouvier (University of Montreal, Montreal, QC).

### Cell culture and transfection

Human embryonic kidney (HEK293) cells and human lung airway epithelial (CALU3) cells, obtained from ATCC (Manassas, VA), were cultured in Dulbecco’s modified Eagles medium (DMEM) supplemented with 10% fetal bovine serum and 1% penicillin streptomycin (Life Technologies, Grand Island, NY). Cells were cultured in humidified 37 °C atmosphere containing 5% CO_2_. HEK293 cells were transiently transfected with 5 µg of N-terminal FLAG epitope tagged β2AR, or the Cys341Ala mutant, with LipoD293 (Signagen Laboratories, Gaithersburg, MD) following manufacturer’s instructions. Whole cell binding with dominant negative dynamin K44A was performed with the addition of 10 µg of dynamin K44A mutant. For β-arrestin studies, HEK293 cells were transiently transfected with 1 µg FLAG-β2AR and 3 µg β-arrestin-2-YFP. All experiments were performed two days post transfection.

### Induction of redox models for functional analysis

The native receptor state was untreated while the oxidized state was induced by incubation of cells with 100 μM H_2_O_2_ for 1 minute immediately prior to drug treatment, as we have shown that this condition stimulates significant Cys-S-sulfenation^21^. The redox deficient receptor state was induced by incubation of cells with 100 μM H_2_O_2_ for 1 minute, removal of oxidant and subsequently incubation with 1 mM dimedone for 15 minutes immediately prior to drug treatment.

### Dimedone and DYn-2 Labeling

HEK-β2AR cells were treated with 5 mM dimedone or DYn-2 (2% v/v DMSO) as previously described^17^ for 1 hour at 37 °C with frequent agitation. Cells were washed with PBS and lysed in modified RIPA (50 mM triethanolamine pH 7.4, 150 mM NaCl, 1% NP-40, 1% Sodium deoxycholate, 1% SDS) supplemented with 2,000 units/mL catalase and cOmplete EDTA-free protease inhibitors for 20 minutes on ice with frequent vortexing. Insoluble debris was removed via centrifugation at ca. 15,000 x g for 15 minutes at 4 °C. For dimedone treated cells, lysates were subject to methanol precipitation as previously described^22^. Briefly, lysates were mixed with 4:4:1 lysate:methanol:chloroform and then centrifuged at ca. 15,000 x g for 20 minutes at 4 °C. The protein was washed with 1:1 methanol:chloroform and centrifuged at ca. 15,000 x g for 10 minutes at 4 °C. Protein was then dried and dissolved in Laemmli sample buffer with 5% SDS and 5% β-mercaptoethanol (βME) and incubated at room temperature for 20 minutes prior to SDS-PAGE. DYn-2 treated lysates were precleared of endogenously biotinylated proteins with 75 µL of Neutravidin beads (Pierce) for 1 hour at room temperature. Purified recombinant human GAPDH from Abcam (ab82633) was incubated with dimedone or DYn-2 as described for whole cells. The labeling was stopped by direct methanol precipitation as described above. The dimedone labeled GAPDH was then resolved in Laemmli sample buffer and boiled for 5 minutes. DYn-2 precipitated protein was re-dissolved in modified RIPA.

### Agonist mediated dimedone labeling in CALU3 cells

CALU3 cells were grown to confluence and cells were treated with or without 1 μM ISO for 5 or 15 minutes prior to incubation with 5 mM dimedone for 15 minutes. Cells were washed with iced PBS and lysed in RIPA and cleared of insoluble debris as described above. The protein lysate (200 μg)was diluted with 900 μL dilution RIPA (25 mM Tris-HCl pH 7.6, 150 mM NaCl, 0.5% NP40) and tumbled with 4 μg anti-human beta-2 adrenergic receptor rabbit polyclonal antibody (H-73 SCBT) or 2-thiodimedone for 2 hours at 4 °C. 35 μL of A/G-agarose beads (Thermo Fischer Scientific) were added and tumbled overnight at 4 °C, beads were washed three times with PBS and protein was eluted with Laemmli with 2.5% βME at room temperature for 20 minutes and were immunoblotted.

### Immunoprecipitation of FLAG-β2AR

50 μL of Dynabeads (Thermo Fischer Scientific) were bound with anti-FLAG M2 antibody (5 µg for 10 minutes) prior to incubation with precleared lysates for 10 minutes at room temperature. Beads were washed three-times with PBS with 0.02% Tween-20 and resuspended in modified RIPA.

### Click chemistry

Dyn-2 labeled protein was incubated with 100 μM azide-biotin, 1 mM tris(2-carboxyethyl)phosphine hydrochloride (TCEP-HCl), 100 μM tris[(1-benzyl-1H-1,2,3-triazol-4-yl)methyl]amine ligand (TBTA) and 1 mM CuSO_4_ at room temperature for 1 hour with agitation. The reaction was quenched with 40 mM ETDA. Whole cell lysates were then subject to methanol precipitation as described above. Immunoprecipitated proteins were washed three-times with modified RIPA and eluted at room temperature for 20 minutes in Laemmli sample with 5% SDS and 5% βME.

### Radioligand binding and competition assays

HEK-β2AR were pelleted by centrifugation at 1,000 x g for 10 minutes. Cells were re-suspended in assay buffer (7.5 mM Tris-HCl and 5 mM EDTA, pH 7.0) and homogenized 20 times. Membranes were pelleted via centrifugation at 40,000 x g for 30 minutes at 4 °C and re-suspended in assay buffer at 1 µg/µl protein with DC Protein Assay (BioRad Laboratories, Hercules CA). For competition binding assays, 50 µg of membrane was incubated with 0.8 nM of [^3^H]-Dihydroalprenolol (Perkin Elmer, Waltham, MA) and varying concentrations of isoproterenol in the presence or absence of 100 µM H_2_O_2_ and/or 1 mM dimedone. Non-specific binding was determined as the amount of radioligand bound in the presence of 10 μM propranolol. 4 mM magnesium chloride was added to the binding buffer to elucidate the effects of divalent cations. CALU3 membranes were prepared in the same manner in 25 mM HEPES with 5 mM EDTA (pH 7.0). Saturation isotherms were used to quantify the maximal binding (B_max_) using approximately 100 µg of protein and a saturating concentration of [^3^H]-Dihydroalprenolol (10 nM) with or without 1 mM H_2_O_2_ and/or 1 mM dimedone, as noted in the figure legends. All reactions were incubated for 30 minutes at room temperature with agitation and terminated by rapid filtration over Whatman filters, washed with iced assay buffer supplemented with 0.1% BSA and assessed by liquid scintillation counting.

### Whole cell radioligand binding assays

300,000 cells were seeded onto a 24 well plates and allowed to adhere overnight. Whole cell binding was performed in DMEM supplemented with 20 mM HEPES (pH 7.2) at 37 °C for 15 minutes. Cells were incubated with approximately 2 nM [^3^H]-Dihydroalprenolol with or without 100 µM H_2_O_2_ and/or 1 mM dimedone. After incubation, cells were immediately placed on ice and washed 5x with ice-cold PBS. Trypsin was used to detach cells and bound radioligand was quantified via liquid scintillation counting.

### ERK1/2 and CREB phosphorylation

HEK-β2AR cells were seeded at 1 × 10^6^ cells per well, allowed to adhere overnight, and then serum starved for 4 hours. CALU3 cells were grown to 80–90% confluence and serum starved for 48 hours. Experiments were performed in fresh DMEM lacking serum. Following treatment with 1 µM ISO or 3 µM forskolin for 5 or 30 minutes, cells were washed 3x with iced PBS and lysed in RIPA buffer (50 mM Tris–HCl, 150 mM NaCl, 1% nonidet P40, 1% sodium deoxycholate, 0.1% SDS, 5 mM EDTA, 10 mM NaF, 1 mM Na_3_VO_4_, pH 7.4) for 20 minutes on ice. Insoluble cellular debris was removed by centrifugation at ca. 15,000 x g and protein levels were standardized using the DC Protein Assay. Lysates were denatured by boiling in Laemmli sample buffer with 2.5% βME for 5 minutes, resolved by SDS-PAGE, and immunoblotted as described below.

### Immunoblotting

Following resolution of total protein by SDS-PAGE and transfer to PVDF membranes, membranes were blocked for 1 hour at room temperature or overnight at 4 °C and washed in TBST prior to primary antibody incubation for 1 hour at room temperature or overnight at 4 °C. Membranes were then washed three-times with TBST and incubated with HRP-conjugated superclonal antibodies from Invitrogen (Carlsbad, CA) (1:15-20,000) and washed again three-times in TBST and imaged by ECL. Membranes were stripped and reprobed with other antibodies as show, and as a result, actin loading control blots from the same blot may be shown in multiple figure panels. Sources and concentrations of antibodies were: Anti-Flag M2 antibody was from Sigma–Aldrich (St. Louis, MO) (1:2-3,000), β-actin (SC4778) (1:1,000), Human β2AR H-73 (SC 9042) (1:500-1,000) and GAPDH (SC25778) (1:1,000) were from Santa Cruz Biotechnology (Dallas, TX); pCREB ser133 rabbit mAB (9198) (1:1,000) and pERK ser42/44 rabbit mAB (4370) (1:1,000) were from Cell Signaling Technologies (Danvers, MA); streptavidin-HRP (21126) (1:25,000) was from Thermo Fischer Scientific (Waltham, MA); Cysteine Sulfenic Acid (2-Thiodimedone) (EST022) was from Kerafast (Boston, MA) (21).

### cAMP Formation

1.25 × 10^5^ cells were seeded to 24 well plates and allowed to adhere overnight. Cells were incubated with 100 µM 3-isobutyl-1-methylxanthine (IBMX) for 5 minutes prior to stimulation for 5 minutes with ISO or 3 μM forskolin. cAMP formation was detected using Cayman Chemical Cyclic AMP ELISA following the manufactures instructions. For assessment of Gαi signaling, cells were treated with 1 μg/ml of pertussis toxin in serum free DMEM for 18 hours prior to assay. Due to the nature of the transient transfections used in HEK- β2AR cells in conjunction with the inter-assay variability derived from ELISA, we analyzed all cAMP with reference to the maximum inducible cAMP response seen with 1 μM ISO. Intra-assay trends were very consistent despite the inter-assay variability in raw cAMP concentration.

### β-arrestin recruitment

Transfected cells were adhered to glass coverslips treated with rat-tail collagen in 6-well plates and were serum-starved for 2 hours prior to a 15 minute stimulation with 10 µM ISO. Cells were washed three-times with iced PBS and fixed with 4% paraformaldehyde. Confocal images were obtained on a Leica TCS SP8 with 40X oil immersion.

### Receptor internalization

β2AR internalization was assessed as we have previously described^10^. HEK-β2AR cells were seeded at 5 × 10^5^ cells per well on 12 well plates and allowed to adhere overnight. Cells were stimulated 10 μM ISO or vehicle for 40 minutes. For inhibition studies, cells were pre-treated with 10 μM propranolol for 5 minutes. Following stimulation, cells were kept at 4 °C for the remainder of the preparation. Cells were washed with PBS and incubated at 4 °C for 1 hour with anti-FLAG antibodies (1:1000 in PBS) with gentle agitation. Cells were washed with PBS and then incubated with FITC-conjugated anti-mouse secondary for 1 hour at 4 °C (1:5,000 in PBS). Cells were washed with PBS and evaluated for FITC fluorescents via flow cytometry. 50,000 events per sample, which approximates to 9,000 gated cells, were analyzed. Internalization was determined by a decreased in mean FITC fluorescence and data are standardized to a percent of propranolol blocked receptor internalization.

### ROS detection

ROS detection was performed as we have previously described^10^. Briefly, CALU3 cells were seeded onto collagenized coverslips and allowed to grow to confluence. Cells were incubated with 10 µM CM-H_2_DCFDA in PBS at 37 °C for 1 hour. CM- H_2_DCFDA was removed and cells were treated with either 1 mM H_2_O_2_ or 10 µM ISO in PBS for 5 minutes at 37 °C, 10 μM alprenolol was added 5 minutes prior to ISO. Cells were washed three-times in iced PBS and fixed with 4% paraformaldehyde at 4 °C for 1 hour. Confocal images were obtained on a Leica TCS SP8 with 40X oil immersion.  Where apocynin (100 µM) or diphenyleneiodonium chloride (10 µM) were used, treatments were performed for two hours in HEPES-buffered DMEM without serum prior to DCFDA loading and ISO treatment as above. 

### Cell viability

Cells were seeded and treated as preformed in the cAMP assay, except IBMX was not included. Following treatment, cells were trypsinized, stained with trypan blue and counted by hemocytometer.

### Data expression and statistical analysis

Images were quantified using NIH Image J software. Integrated density for immunoblots was standardized to the loading and evaluated as a percent of the isoproterenol stimulated response in the presence or absence of. Data are expressed as mean ± S.E.M for representative experiments repeated at least three independent times. Given the differences in absolute values of raw data due to transient transfection of HEK-β2AR cells, these were normalized to the respective percent control. Where not visible, error bars fall within the symbol size. Statistical analysis was performed, as appropriate, either by one-way analysis of variance and post-hoc Tukey’s test or by unpaired *t*-test using Graphpad Prism or Microsoft Excel and are represented as *p < 0.05, **p < 0.01, ***p < 0.001. Second and third comparisons are performed using a similar classification with the † and # symbols, as noted in the figure legends.

## Results

### *In situ* β2AR Cys-S-sulfenation

**Figure 1:**
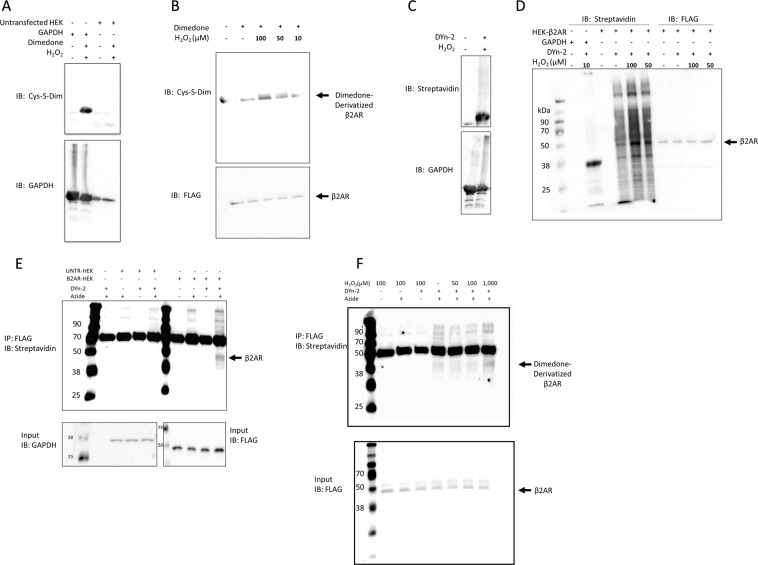
β2AR is oxidized by H_2_O_2_ and can be subsequently alkylated by dimedone/DYn-2 *in situ*. (**A**) Oxidation of purified recombinant human GAPDH was assessed as a positive control and validation for the methodology. GAPDH was exposed to 10 μM H_2_O_2_ and labeled with dimedone for 1 hr, followed by SDS-PAGE and immunoblotting with an anti-Cys-S-dimedone antibody. Untransfected HEK-β2AR cells were also assessed to validate specificity. (**B**) *In situ* oxidation of β2AR occurs upon treatment with H_2_O_2_ in a concentration-dependent manner. HEK-β2AR cells were treated with H_2_O_2_ and/or dimedone as shown, cells were lysed, and proteins resolved by SDS-PAGE then immunoblotted with an anti-Cys-S-dimedone antibody (upper). The immunoreactive band at approximately 48 kDa corresponds to the size of β2AR and aligns with the FLAG-M2 immunoreactive protein band (lower) to demonstrate equal expression and loading of β2AR (n = 4). (**C–E**) The alkyne-containing dimedone analog DYn-2 alkylates Cys-S-sulfenic acids on purified GAPDH and β2AR from HEK-β2AR *in situ*. In (**C**), purified GAPDH was exposed to 10 µM H_2_O_2_ and click chemistry was used to biotinylate the probe as described in the materials and methods. Following SDS-PAGE, proteins were immunoblotted with streptavidin-HRP to detect DYn-2-biotin containing label (upper), while total GADPH is shown to demonstrate equal protein loading (lower). In (**D**), H_2_O_2_ concentration-dependent cell-wide Cys-S-sulfenation is visualized using the same approach as in (**C**). The FLAG-immunoreactive band at 48 kDa, corresponding to β2AR (right), aligns with a 48 kDa protein that displays concentration-dependent streptavidin signal in the presence of H_2_O_2_ (center) (n = 3). (**E**) DYn-2 and Click chemistry control conditions in untransfected cells and those with transfected with FLAG- β2AR. (**F**) Immunoprecipitated β2AR is oxidized by Cys-S-sulfenation is dependent on oxidant concentration (upper). The ~48 kDa β2AR band is seen underneath the thick IgG band. Total FLAG-β2AR expression (lower) is shown to demonstrate equivalent protein expression and loading (n = 3).

Using a modified-biotin switch experiment, we have previously demonstrated that agonist-mediated ROS generation or exposure to exogenous ROS in the form of H_2_O_2_ can elicit Cys-S-sulfenation of the β2AR protein^21^. Here, we sought to determine whether β2AR can be Cys-S-sulfenated by oxidants *in situ*. To begin to do so, we utilized the small molecular diketone dimedone, a selective and irreversible alkylator of cysteine S-sulfenic acids^14,15,23,24^, which covalently labels these oxidized residues^21,25^. Upon incorporation of dimedone, these Cys residues can then be detected by immunoblotting with an antibody targeting dimedone-bound cysteine (2-thiodimedone) residues^26^. To determine the validity of this approach, we utilized purified GAPDH, which is known to be Cys-S-sulfenated and labeled by dimedone^15,16,23^, as an experimental positive control and indeed, a dimedone reactive band corresponding to Cys-S-sulfenated GAPDH was observed (Fig. 1A). Using this approach, untransfected HEK293 cells revealed no immunoreactivity (Fig. 1A), while HEK293 cells transiently expressing FLAG-epitope tagged β2AR (HEK-β2AR) showed concentration-dependent H_2_O_2_ dimedone-labeling (Fig. 1B). To confirm the oxidant-sensitive reactivity was not secondary to altered FLAG-β2AR expression, we immunoprecipitated β2AR via the FLAG-epitope and assessed expression by immunoblotting. As expected, treatment did not affect FLAG-β2AR expression in transfected cells and immunoreactivity was absent in untransfected cells (Supplementary Fig. S1). These data demonstrate that the β2AR is Cys-S-sulfenated *in situ*, and that *in situ* β2AR Cys-S-sulfenic acids can be alkylated by dimedone.

To validate these results, we utilized a different labeling approach that made use of the alkyne-containing dimedone analog DYn-2, which also has high affinity and selectively toward Cys-S-sulfenic acids^17^, but can be biotinylated post-lysis via click chemistry and detected by streptavidin-based ECL. Our results with GAPDH validate the efficacy of DYn-2 in labeling and detection of Cys-S-sulfenic acids (Fig. 1C). In whole cell lysates from HEK-β2AR cells, DYn-2 detected abundant basal genomic wide Cys-S-sulfenation, as previously reported^13,14,20–22^, and this effect was significantly increased in a concentration-dependent manner by treatment with H_2_O_2_ (Fig. 1D). Further, a 48 kDa sized thick band of immunoreactivity corresponding to the expected size and intensity of overexpressed β2AR demonstrated significant Cys-S-sulfenation as detected by this whole-cell lysate approach (Fig. 1D; FLAG reactive bands from darker exposure; single exposure from same blot shown as Supplementary Fig. S2). To elucidate the exact β2AR specific Cys-S-sulfenic acid labeling, we immunoprecipitated β2AR using anti-FLAG antibody from cells incubated with DYn-2 and/or H_2_O_2_ *in situ*. Immunoprecipitated protein was enzymatically digested with trypsin and chymotrypsin and confirmed to be the β2AR protein by electrospray ionization/mass spectrometry (data not shown) and 1/10 of the eluate was also immunoblotted for β2AR to confirm on a gel, as in Supplementary Fig. S1. Subsequently, we utilized click chemistry to biotinylate DYn-2 and then detected DYn-2-biotinmodified β2AR with streptavidin-based ECL. Control conditions reveal the detection of β2AR with basal DYn-2 incorporation in the absence of exogenous H_2_O_2_ (Fig. 1E–F). Incubation in the presence of H_2_O_2_ significantly increased the incorporation of Dyn2 in the presence of 1 mM H_2_O_2_. Taken together, our data confirm that the β2AR is Cys-S-sulfenated and susceptible to labeling by dimedone based probes *in situ*. Of note, both the whole cell *in situ* labeling with dimedone (Fig. 1B) or Dyn-2 (Fig. 1D–F) reveal the presence of basal levels of labeling in the absence of added H_2_O_2_, indicative of some degree of constitutive oxidation, as well as an increase in that level upon treatment with exogenous oxidant.

#### Oxidation of β2AR increases the number of available orthosteric binding sites

**Figure 2:**
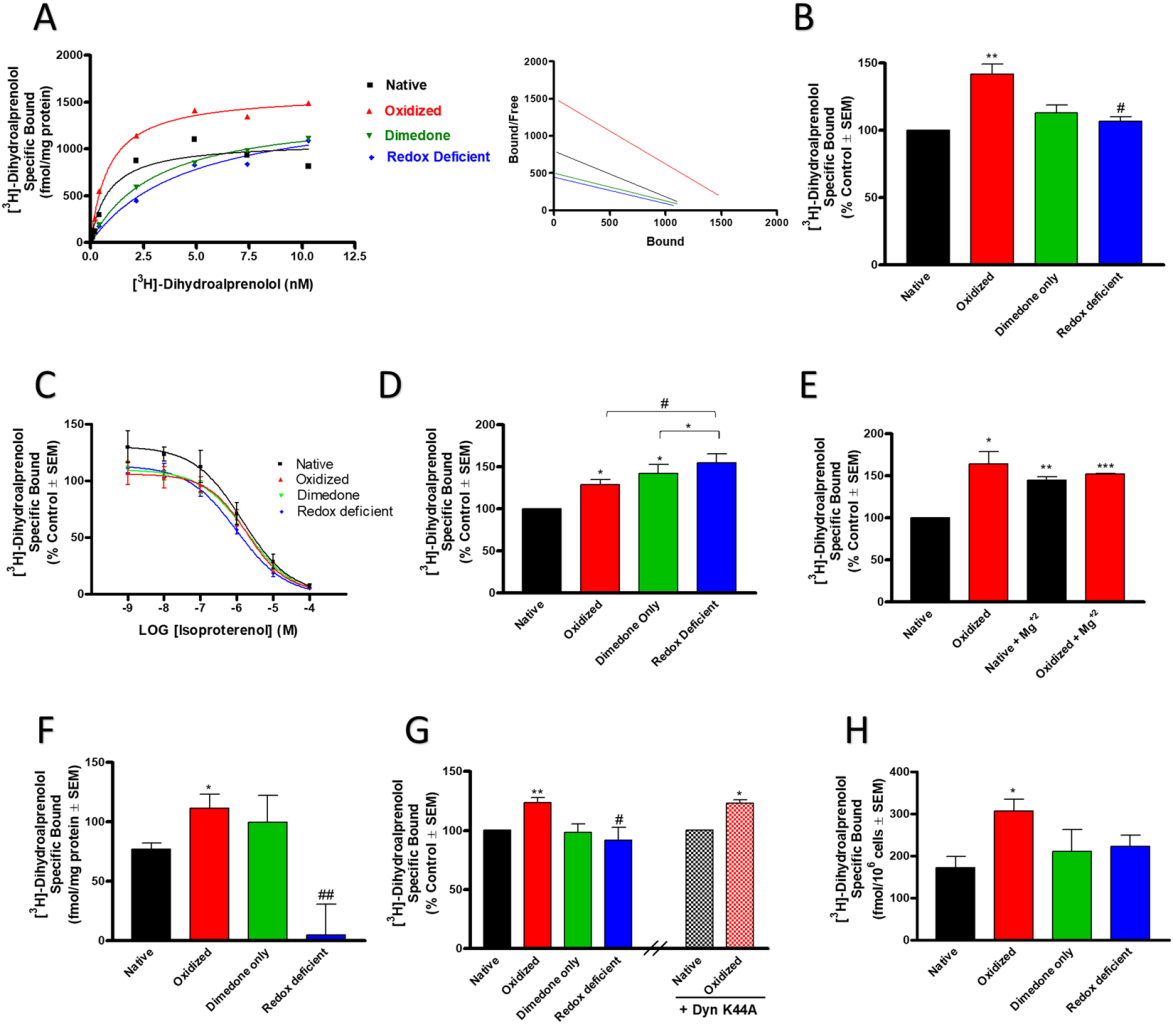
Oxidation of β2AR increases the total number of available orthosteric binding sites. (**A**) Saturation binding of [^3^H]-dihydroalprenolol to HEK-β2AR membranes reveals an increase in the B_max_ in the oxidized state, an effect that is signficantly reversed by alkylation of sulfenic acids by dimedone in the redox-deficient state (left) (n = 3). Scatchard analysis reveals B_max_ of 1296 and 1266 fmol/mg protein in the native and redox-deficient states, respectively, and 1702 fmol/mg protein in the oxidized state (right). (**B**) A saturating concentration (10 nM) of [^3^H]-dihydroalprenolol was used for further experiments and shows significant increases in B_max_ (141.6 ± 12.9% of control) compared to control (*p* < 0.01), while the redox-deficient state exhibits significantly decreased B_max_ (106.5 ± 5.9%), compared to the oxidized state (*p* < 0.05) (n = 3). (**C**) Competition binding of [^3^H]-dihydroalprenolol with isoproterenol reveals that [^3^H]-dihydroalprenolol can be fully displaced by ISO in all redox states demonstrating that redox affects the orthosteric binding site (n = 4). (**D**) The B_max_ of [^3^H]-dihydroalprenolol was also significantly increased (128.8 ± 5.6%) by oxidation in β2AR-C341A expressing membranes versus native controls (*p* < 0.05), however, dimedone alone (142.2 ± 10.3%; *p* < 0.05 vs native) or following oxidation (154.3 ± 11.1%; *p* < 0.05 vs native) further increased B_max_ in these membranes, suggesting involvement of other cysteine residues (n = 4). (**E**) The oxidation-mediated increases in B_max_ (164.0 ± 14.8%) could be mimicked by the presence of Mg^+2^ (144.6 ± 4.2%), but not further increased by it (152^.2 ± ^0.8%) (n = 2). (**F**) A saturating concentration (10 nM) of [^3^H]-dihydroalprenolol revealed significant increases in B_max_ (111.4 ± 11.5 fmol/mg protein) compared to control (76.6 ± 5.6 fmol/mg protein (*p* < 0.0.5) in isolated plasma membranes from human lung airway epithelial (CALU3) cells that endogenously express β2AR; while the redox-deficient state exhibits near abolishment of ligand binding, (B_max_ of 4.5 ± 26 fmol/mg protein), compared to the oxidized state (*p* < 0.01) (n = 5). (**G**,**H**) Ligand binding alterations persisted in live HEK-β2AR cells in a manner independent of receptor internalization, as noted by the ability of oxidized states expressing dynamin K44A (123.3 ± 2.7%) to display equivalent responses to oxidation alone (123.7 ± 4.2%), as well as in live CALU3 cells (H). ^*^And ^**^denote *p* < 0.05 and *p* < 0.01, respectively, versus native condition. ^#^And ^##^denote *p* < 0.05 and *p* < 0.01, res*p*ectively, versus oxidized condition.

Given that dimedone and DYn-2 were shown to be incorporated into oxidized β2AR cysteine residues, and that this modification is known to be covalent^17,18^, we assessed the consequences of receptor oxidation using three oxidative states of the receptor. In these studies, the native state of the receptor, with normal redox cycling capability is compared to the oxidized state that is induced by H_2_O_2_ (100 µM for 1 minute), as shown previously^21^ and in Fig. 1. However, in the presence of dimedone, β2AR Cys-S-sulfenic acids are covalently and irreversibly bound by the Cys-S-OH alkylator and become redox-deficient, or incapable of further redox cycling, also as shown previously^7^ and in Fig. 1. We first tested the effects of receptor oxidation and redox deficiency on β2AR ligand binding from isolated plasma membranes from HEK-β2AR cells. Due the transient nature of receptor transfection in these experiments leading to conceivably variable total β2AR expression between experiments (data not shown), all HEK-β2AR results were normalized to the native state control condition. Saturation binding of [^3^H]-dihydroalprenolol demonstrated a significant increase in specific binding upon oxidation with H_2_O_2_, an effect that was reversed by dimedone alkylation, though dimedone alone did not alter ligand binding (Fig. 2A,B). Scatchard analysis revealed a significant increase in the [^3^H]-dihydroalprenolol B_max_ in oxidized states compared to both native and redox-deficient states, however, there was no significant alteration to the binding affinity (K_D_) of the radioligand (Fig. 2A,B; Table 1). Competition binding of ISO versus [^3^H]-dihydroalprenolol revealed that the radioligand could be fully displaced by the agonist in all redox states and that the affinity and Hill slope of ISO binding were unaltered by redox states (Fig. 2C; Table 2). These data suggest that Cys-S-sulfenation of the β2AR may regulate ligand accessibility to the orthosteric binding pocket.

**Table 1:** Binding parameters of [^3^H]-Dihydroalprenolol at HEK-β2AR membranes in native, oxidized, dimedone-alone, and redox-deficient states.

	Native	Oxidized	Dimedone	Redox-Deficient
Bmax (% of Native ± SEM)	100	141.6 ± 12.9	112.9 ± 10.4	106.5 ± 5.9
K_D_ (nM ± SEM)	1.90 ± 0.24	2.36 ± 0.72	2.51 ± 0.21	2.47 ± 0.27

**Table 2:** Binding parameters of Isoproterenol vs. [^3^H]-Dihydroalprenolol at HEK-β2AR membranes in native, oxidized, dimedone-alone, and redox-deficient states.

	Native	Oxidized	Dimedone	Redox-Deficient
K_i_ (nM ± SEM)	951 ± 362	1250 ± 626	838 ± 381	702 ± 268
n_H_ ± SEM	−0.66 ± 0.06	−0.8 ± 0.05	−0.67 ± 0.11	−0.65 ± 0.06

Since Cys341 palmitoylation is known to be crucial for β2AR ligand binding^27^, we sought to determine if the redox-dependent response of receptor oxidation persisted in the absence of palmitoylation by utilizing a β2AR-C341A mutant. These results show that H_2_O_2_-mediated oxidation of β2AR-C341A also increases the B_max_ of [^3^H]-dihydroalprenolol (Fig. 2D), albeit to a lower total extent than wild type β2AR (data not shown), however, the dimedone-only control and redox-deficient states exhibited even greater increases in ligand binding (Fig. 2D). These data demonstrate that the effects of Cys-S-sulfenation are not limited to Cys341, and that trapping of redox-active cysteines with dimedone induces different alterations to the binding pocket in the absence of palmitoylation. Importantly, since β2AR-C341A is known to significantly alter receptor affinity^24^, this result suggests that the positive-regulation of the orthostatic site persists in the low affinity state. Since mono and divalent cations, such as Na^+^, Mg^+2^, and Zn^+2^, are known to act allosterically to affect the B_max_ of GPCR ligands, including for β2AR^28,29^, we aimed to evaluate if receptor oxidation implored a similar effect to that seen with Mg^+2^. HEK-β2AR membranes incubated in the presence of 4 mM Mg^+2^ or 100 μM H_2_O_2_, alone or in combination, increased [^3^H]-dihydroalprenolol binding, however the effect was not additive (Fig. 2E), demonstrating β2AR S-sulfenation can mimic the effect of cations, plausibly through a redundant allosteric binding mechanism.

We next sought to determine if the effects seen in HEK293 cells persisted in physiologically relevant cells that endogenously express β2AR. Hence, we utilized the human lung airway epithelial cell model (CALU3) and performed membrane binding studies with saturating concentrations of [^3^H]-dihydroalprenolol, as above. Oxidation of CALU3 membranes with H_2_O_2_ also significantly increased [^3^H]-dihydroalprenolol binding (Fig. 2F), as was seen in HEK-β2AR membranes. In these cells, the redox-deficient state induced by H_2_O_2_ followed by dimedone trapping demonstrated near abolishment of [^3^H]-dihydroalprenolol binding, while dimedone alone had no effect (Fig. 2F). These results demonstrate that oxidation of β2AR positively regulates ligand binding in airway epithelial cells and that the redox-deficient state of the receptor fails to appropriately bind ligand. Furthermore, β2AR oxidation state elicits the same trend of ligand binding in CALU3 cells as HEK- β2AR, but with an exaggerated response seen in the more physiologically relevant cell line.

Lastly, to determine that these membrane binding results translated to live cells, we performed whole-cell binding assays in HEK-β2AR and CALU3 cells. As seen in membranes, oxidation of HEK-β2AR cells elicited significant increases in whole-cell [^3^H]-dihydroalprenolol binding, an effect that was lost in the redox-deficient state (Fig. 2G). To ensure that these effects were not simply due to the ability of oxidation to alter receptor internalization, which would bring to light heightened [^3^H]-dihydroalprenolol binding, we repeated the experiments in cells that co-expressed the dominant-negative dynamin K44A mutant, which is incapable of internalizing β2AR, as we have shown previously^30^. In cells expressing Dyn-K44A, oxidation increased [^3^H]-dihydroalprenolol to the same degree as seen in HEK-β2AR cells, demonstrating that the effects of oxidation are independent of β2AR internalization (Fig. 2G). Similarly, oxidation increased [^3^H]-dihydroalprenolol binding to endogenous β2AR on live CALU3 cells, and this effect was seemingly reversed in the redox-deficient state, though it was not significant (*p* = 0.09) (Fig. 2H). Taken together, these results demonstrate that oxidation exposes additional orthosteric ligand binding sites in membranes and live cells, and that Cys-S-sulfenic acid trapping by dimedone renders these sites inaccessible.

#### Redox active cysteines modulate canonical cAMP responses

**Figure 3:**
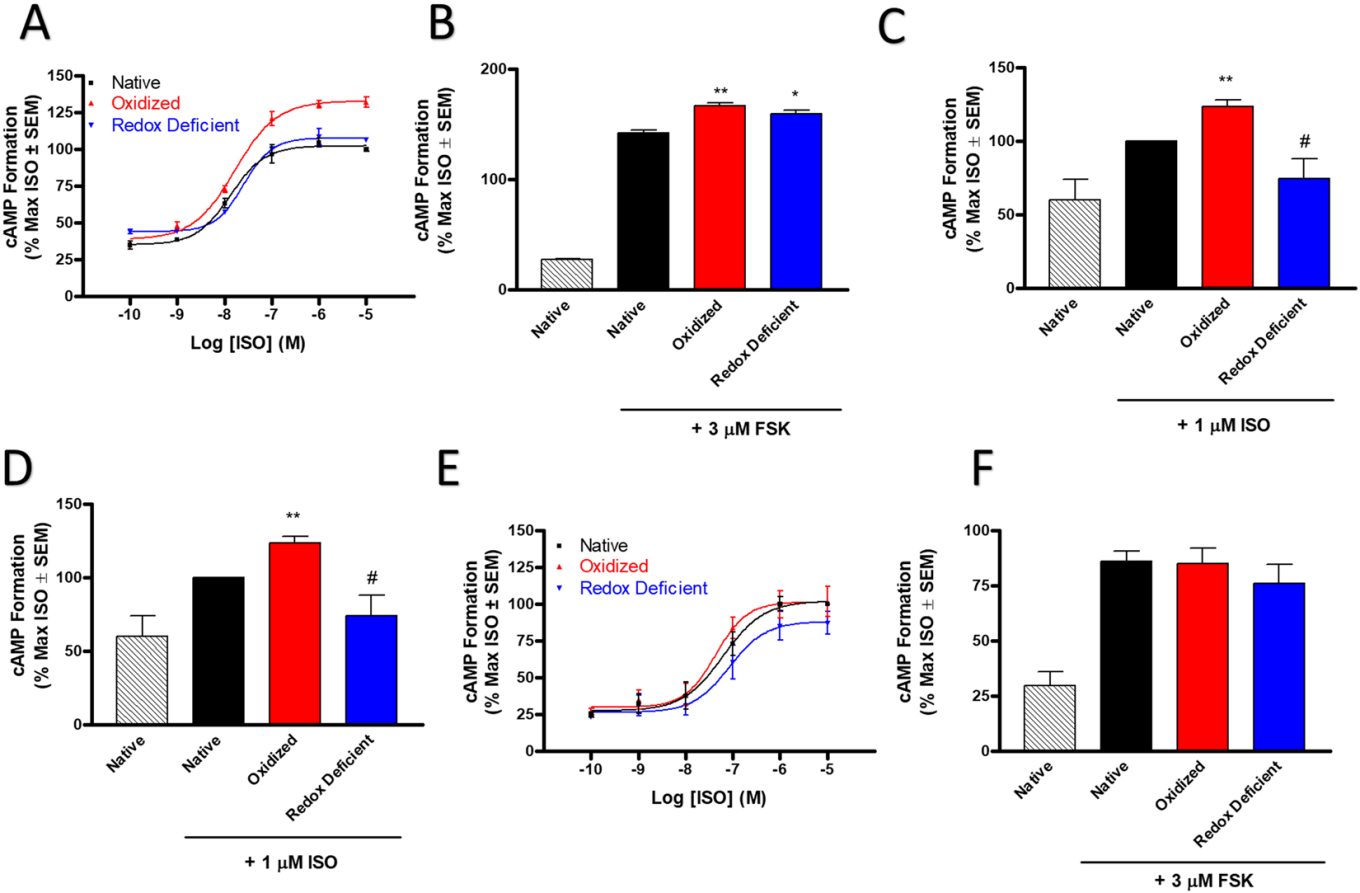
Redox active cysteine residues are necessary for canonical β2AR-mediated cAMP formation. (**A**) Oxidized HEK-β2AR cells exhibit a significantly heightened ISO-induced cAMP response versus the native state (*p* < 0.001 via ANOVA with Tukey post-hoc), and this effect is abolished in the redox-deficient state (*p* < 0.05 via ANOVA with Tukey post-hoc). The peak ISO-induced effect (at 10 µM) was 132.3 ± 3.4% of native control in the oxidized state and 106.5 ± 0.8% of native control in the redox-deficient state (n = 3). (**B**) Oxidation by H_2_O_2_ significantly increases (*p* < 0.01) forskolin (FSK)-stimulated cAMP formation versus native control in HEK-β2AR cells and this effect was not reversed by dimedone in the redox-deficient state (*p* < 0.01 vs native), demonstrating that the effects seen in (**A**) are specificly mediated by β2AR (n = 3). (**C**) Oxidation by H_2_O_2_ significantly increases (*p* < 0.01) ISO-stimulated cAMP formation versus native control in HEK-β2AR-C341A expressing cells and this effect is significantly reversed (*p < *0.05 vs oxidized) by dimedone in the redox-deficient state, demonstrating that the effects seen with ISO are not soley attributed to Cys341 palmitolyation (n = 3). (**D**) Redox-deficient CALU3 cells exhibit a signficantly lower ISO-induced cAMP response versus the native (*p* < 0.05 via ANOVA with Tukey post-hoc) and oxidized states (*p* < 0.01 via ANOVA with Tukey post-hoc) (n = 3). (**E**) Oxidation by H_2_O_2_ did not alter FSK-stimulated cAMP formation versus native control in CALU3 cells and this effect was decreased, though not significantly by dimedone in the redox-deficient state (n = 3). ^*^And ^**^denote *p* < 0.05 and *p* < 0.01, respectively, versus native condition. ^#^Denotes *p* <0.05 versus oxidized condition via un*p*aired *t*-test.

Next, we aimed to determine if the redox sensitive effects on ligand binding translate to alterations in β2AR function. Since agonism of β2AR increases Gαs mediated cAMP formation, we assessed cAMP formation in native, oxidized, and redox-deficient states of the β2AR in whole cells. In HEK-β2AR cells, ISO-stimulated cAMP formation was significantly (*p* < 0.01 ANOVA) increased in the oxidized state, while redox-deficient receptors were significantly (*p* < 0.05 ANOVA) impaired in ability to form cAMP versus the oxidized state (Fig. 3A). At an E_max_ concentration of ISO (1 µM), oxidized receptors showed a 32.3 ± 3.4% increase in cAMP formation above that of native receptors, while blockade of S-sulfenation via converting sulfenic acids to redox-deficient residues by the presence of dimedone abolished this effect (Fig. 3A). Control experiments showed no effects of H_2_O_2_ (i.e., oxidized state), H_2_O_2_ and dimedone (i.e., redox-deficient state) or dimedone alone (Supplementary Fig. S3) on basal cAMP formation in the absence of ISO demonstrating that the effect is specific to ISO-mediated cAMP formation. To ensure that our results are not simply a consequence of redox modification to adenylyl cyclase, we compared the ability of forskolin (FSK) to elicit a cAMP response in native, oxidized, and redox-deficient states. Here, H_2_O_2_ alone significantly increased FSK-induced cAMP formation (25 ± 2.3% above native state), however, unlike that seen with ISO, dimedone treatment did not reverse the effect (Fig. 3B), suggesting that the redox-deficient inhibition of cAMP formation is mediated by β2AR cysteine alkylation. Since Cys341 palmitoylation is vital to β2AR mediated cAMP formation, we assessed cAMP formation of the three redox states in cells expressing β2AR-C341A. Here, agonism with ISO facilitated an increase in cAMP formation, which was significantly increased upon oxidation and was again attenuated by dimedone labeling in the redox-deficient state (Fig. 3C). These results demonstrate that the loss of function response in the redox-deficient state is not merely a result of dimedone effects on Cys341 palmitoylation, but rather at least in part involves other cysteine residues.

Next, we assessed the functional effects of the three β2AR redox states in CALU3 human lung airway epithelial cells. Here, ISO-stimulated cAMP formation was significantly decreased in the redox-deficient state compared to both oxidized (*p* < 0.01 ANOVA) and native receptor states (*p* < 0.05 ANOVA) (Fig. 3D). Similar to HEK-β2AR cells, H_2_O_2_ and H_2_O_2_ and dimedone treatment had no effect on basal cAMP levels in the absence of ISO in these cells (data not shown) and oxidation states had no impact on adenylyl cyclase function as determined by the FSK-induced cAMP responses (Fig. 3E). Since it is known that PKA can uncouple β2AR from Gαs signaling and elicit a switch to Gαi signaling^28^, we utilized pertussis toxin (PTX) to determine if the reduction in cAMP formation seen here was due to PTX-sensitive Gαi coupling switch. Results in HEK-β2AR and CALU3 cells show that PTX treatment had no effect on redox-state dependent ISO-mediated cAMP formation (Supplementary Fig. S4). Finally, we show that the reductions in cAMP formation seen here was not due to cell death as treatment of both cell lines with all modulators used herein did not alter cell viability (data not shown). Taken together, these results corroborate our binding results and demonstrate that redox-deficient receptors have decreased comparative agonist-stimulated functional output that is dependent on canonical β2AR-Gαs signals.

#### Functional impairment in redox-deficient receptors persists downstream

**Figure 4:**
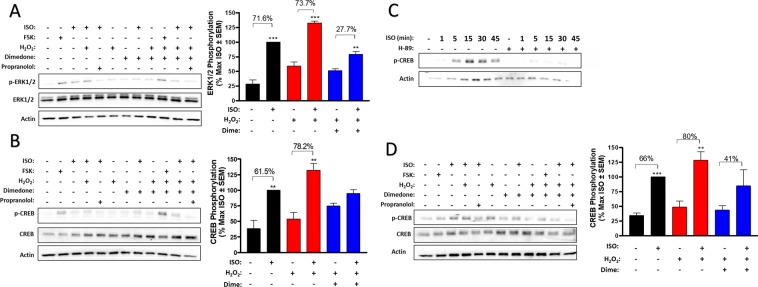
Impaired β2AR signaling from redox-deficient receptors persists to downstream G-protein mediated effectors. (**A**,**B**) In HEK-β2AR cells, ISO stimulates a similar increase in ERK1/2 phosphorylation (**A**) and CREB phosphorylation (**B**) in native and oxidized states, however redox-deficient states have significantly decreased ISO responses. In (**A**), ISO induces a 71.6% and 73.7% increase in ERK1/2 phosphorylation in native (*p* < 0.001 vs native without ISO) and oxidized (*p* < 0.001 vs oxidized without ISO) states, respectively, but only a 27.7% increase in the redox-deficient state (*p* < 0.01 vs redox-deficient without ISO) (n = 3). In (**B**), ISO induces a 61.5% and 78.2% increase in CREB phosphorylation in native (*p* < 0.01 vs native without ISO) and oxidized (*p* < 0.01 vs oxidized without ISO) states, res*p*ectively, but no significant increase was elicited by ISO in the redox-deficient state (n = 3). In CALU3 cells, ISO-induced CREB phosphorylation is mediated by PKA in a temporally transient manner (n = 2). (**D**) In CALU3 cells, ISO induces a 66% and 80% increase in CREB phosphorylation in native (*p* < 0.001 vs native without ISO) and oxidized (*p* < 0.01 vs oxidized without ISO) states, respectively, but only a non-significant 41% increase in the redox-deficient state (n = 4). ** and *** denote *p* < 0.01 and *p* < 0.001, respectively, versus the own redox state control via un*p*aired *t*-test. Full length immunoblots are shown in Supplementary Fig. S5.

To elucidate the effects of β2AR redox state on downstream effectors, we evaluated phosphorylation of ERK1/2 and the cAMP Response Element Binding Protein (CREB). Short-term agonism of HEK-β2AR (1–10 minutes) with ISO has been shown to cause robust ERK1/2 phosphorylation that is solely dependent upon G-protein signaling^31^. Oxidized receptors increased ERK1/2 phosphorylation significantly more than native receptors (*p* < 0.001), and redox deficient receptors had a significantly attenuated ERK1/2 phosphorylation response compared to both native (*p* < 0.05) and oxidized (*p* < 0.001) receptors, however the presence of H_2_O_2_ significantly increased ERK1/2 phosphorylation in the absence of agonist (*p* < 0.05), hence our analysis of ERK1/2 phosphorylation compares each ISO-stimulated condition to its own redox state control (Fig. 4A). Treatment of HEK-β2AR in the native and oxidized states elicited a robust ERK1/2 phosphorylation response to 1 µM ISO (5 minutes), facilitating a 71.6% and 73.7% increase in phosphorylation, respectively (Fig. 4A). Meanwhile, in the redox-deficient state induced by H_2_O_2_ followed by dimedone trapping, the ISO-stimulated ERK1/2 phosphorylation reached only a 27.7% maximal effect (Fig. 4A). In each case, the ISO-stimulated effect was blocked by the β-antagonist propranolol. We also assessed this effect in CALU3 cells, but no receptor-mediated effect on ERK1/2 was detected at this time-point or a longer 30-minute time point plausibly due to the very high basal ERK1/2 phosphorylation in these cells, even upon 48 hours of serum-starvation (data not shown).

To determine if the redox-dependent functional responses seen here modulate downstream transcriptional effects of cAMP, we evaluated the ability of the three β2AR states to phosphorylate the transcription factor CREB^30,31^. In HEK-β2AR cells, stimulation with 1 μM ISO (30 minutes) induced a 61.5% and 78.2% increase in CREB phosphorylation in the native and oxidized states, respectively (Fig. 4B). On the contrary, agonism by ISO was incapable of inducing a significant increase in CREB phosphorylation in the redox-deficient state (Fig. 4B), demonstrating that redox capable cysteines are necessary for β2AR-induced CREB transcriptional activity. As with ERK1/2 phosphorylation, the effects of ISO on CREB phosphorylation were blocked by propranolol in the absence and presence of dimedone and H_2_O_2_ (Fig. 4B). Oxidized receptors again elicited a greater phosphorylation response than native receptors (*p* < 0.05) and oxidation alone also increased CREB phosphorylation, however, this effect was not statistically significant. Since β2AR-mediated CREB phosphorylation is completely uncharacterized in CALU3 cells, we assessed this effect and our results show that ISO-mediated CREB phosphorylation occurs in a temporally transient manner that peaks at 15–30 minutes and is completely mediated by PKA as the effect was abolished by the PKA inhibitor H-89 (Fig. 4C). ISO-mediated phosphorylation of CREB was increased by 68.4% in native CALU3 cells, whereas CALU3 cells in the oxidized state produced an increase of 79.7% (Fig. 4D). Importantly redox-deficient CALU3 cells produced a statistically insignificant increase (ca. 40%) in CREB phosphorylation (Fig. 4D), demonstrating that redox-dependent effects of β2AR are conserved in lung airway epithelial cells. As expected, ISO mediated effects were completely blocked by propranolol in these cells. Oxidized receptors elicited a significantly increased CREB phosphorylation response compared to native receptors (*p* < 0.05), however as in HEK-β2AR cells, oxidation alone also significantly increased CREB phosphorylation to a similar relative amount (*p* < 0.05), hence, our analysis is comparative within each stimulated condition’s own redox state control.

#### β-arrestin mediated signaling is attenuated in redox-deficient states

**Figure 5:**
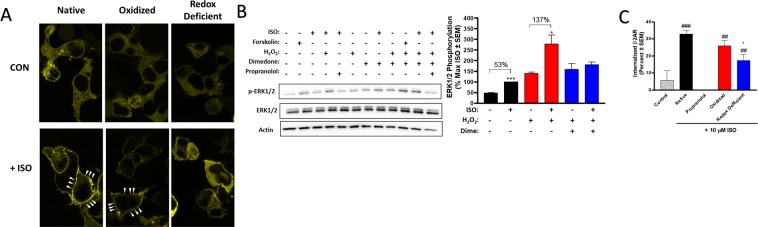
Redox active cysteine residues are also necessary β2AR functional responses mediated by β-arrestin. (**A**) Agonism of HEK-β2AR cells expressing β-arrestin-2-YFP with 10 µM ISO for 15 min stimulates robust translocation of β-arrestin-2-YFP to the plasma membrane in both native and oxidized states, whereas β-arrestin-2-YFP remains mainly cytosolic in the redox-deficient state, as visualized by confocal microscopy. (**B**) In the native state, ISO-stimulates a 53% increase in ERK1/2 phosphorylation at 30 minutes following agonism, a time-point known to be mediated by β-arrestin in HEK-β2AR cells (ref. ^26^). Receptor oxidation significantly enhances this effect (137%), while redox-deficient receptors were unable to evoke a significant β-arrestin-mediated ERK1/2 phosphorylation response upon agonism with ISO (n = 3). The full length immunoblot is shown in Supplementary Fig. S6. (**C**) In the native state, agonism with 10 µM ISO for 40 min results in internalization of 33 ± 2% of β2AR, an effect that is completely blocked by pretreatment with 10 µM propranolol, and which is significantly decreased to 17 ± 4% in the redox-deficient state (*p* < 0.05 versus native state) (n = 3). ^*^Denotes *p* < 0.05 versus native state. ^##^And ^###^denote *p* < 0.01 and *p* < 0.001, respectively, versus the propranolol-treated condition, via unpaired *t*-test.

To assess the effects of Cys-S-sulfenation on β2AR-β-arrestin signaling, we examined ISO-induced β-arrestin-2-YFP recruitment following agonism of β2AR via confocal microscopy. While redox state alone in the absence of agonist had no impact on β-arrestin-2-YFP localization, agonism with 10 µM ISO (15 minutes) induced profound translocation of β-arrestin-2-YFP to the plasma membrane in both native and oxidized states (Fig. 5A), while on the contrary, β-arrestin-2-YFP remained cytosolic in the redox-deficient state (Fig. 5A). To study β2AR-β-arrestin-2 signaling, we examined the sustained agonist-mediated phosphorylation of ERK1/2, which has been shown to be fully β-arrestin-dependent following 10–60 minutes of agonism^31^. Our data, as others have shown previously^32–34^, demonstrates an increase in ERK1/2 phosphorylation following treatment with H_2_O_2_. Omitting the oxidant-induced increase in ERK1/2 phosphorylation described by others^32–34^ and accounting for only the ISO-mediated effect, reveals that agonist-stimulated ERK1/2 phosphorylation was redox-state dependent. Here, oxidized receptors elicited a drastically larger (137% increase) ISO-stimulated ERK1/2 phosphorylation response compared to native receptors (53% increase), while redox-deficient receptors were unable to elicit a significant ISO-mediated ERK1/2 response (Fig. 5B). Finally, since agonism of β2AR with ISO causes β-arrestin-dependent receptor internalization within a timeframe of 30–40 minutes in HEK293 cells^35–38^, we assessed the role of cysteine redox states on internalization. Following a 40 minute stimulation with 10 µM ISO, approximately 40% of FLAG-tagged β2ARs internalized, and this effect was blocked by 10 µM propranolol, as we have also previously reported^30^ (Fig. 5C). Receptor oxidation did not have a significant impact on internalization, however redox-deficient receptors had significantly reduced ability to internalize compared to native receptors (Fig. 5C). Receptor oxidation state in the absence of ISO had no significant impact on receptor localization to the plasma membrane (data not shown). Together, these data indicate that redox-deficient receptors exhibit attenuated β-arrestin recruitment and β-arrestin-dependent signaling and functional outcomes.

#### β2AR agonism induces ROS generation in CALU3 cells that can be modified by Dimedone *in situ*

**Figure 6:**
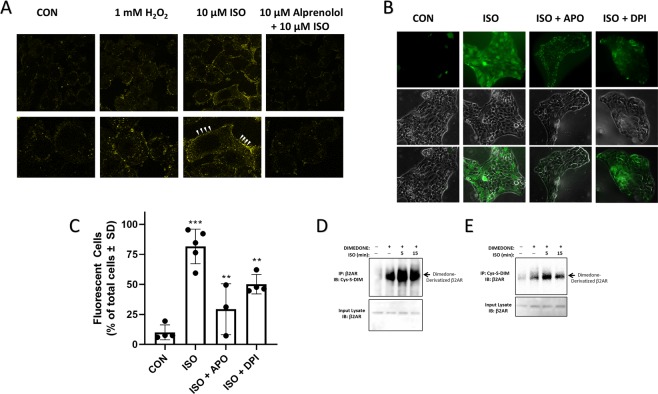
Agonism of β2AR on human lung airway epithelial cells stimulates ROS generation. (**A**) CALU3 cells were preloaded with the intracellular ROS-sensitive fluorescent probe CM-H_2_DCFDA (10 µM) and stimulated with 10 μM ISO for 5 min or as otherwise shown, prior to fixation and confocal imaging. ISO stimulation elicited robust fluorescence, indicative of ROS generation, at the cell membrane, and this effect was blocked by pretreatment with alprenolol (10 µM). The overall degree of fluorescence was equivalent to that seen upon exogenous treatment with 1 mM H_2_O_2_. The upper panel is 40X image and the lower panel depicts further zoom on a single cell from that image. (**B**) Fluorescent microscopy of cells that were pretreated with the NADPH oxidase inhibitors apocynin (APO; 100 µM) and diphenyleneiodonium chloride (DPI; 10 µM) reveals marked decreases in the fluorescent intensity (**B**) and total number of fluorescent cells (**C**) in APO and DPI treated states. Images in (**B**) represent a 20X objective image capture with the top image representing FITC fluorescence, the middle image representing the respective transilluminated image, and the lower image representing the overlay. (**D**,**E**) Agonism of CALU3 cells with 1 μM ISO for 5 or 15 minutes oxidized cysteine residues on the β2AR enabling selective dimedone alkylation of these sulfenic acid residues with 5 mM dimedone (15 minutes) as can be seen by either β2AR immunoprecipitation followed by anti-Cys-dimedone immunoblotting (**D**) or Cys-dimedone immunoprecipitation followed by β2AR immunoblotting (**E**). Full length immunoblots are shown in Supplementary Fig. S7.

Agonist-mediated β2AR ROS generation has been reported in a variety of cells and tissues, but to our knowledge, has not been demonstrated in any airway epithelial cell. Given that our results here demonstrate significant effects of redox on β2AR function in CALU3 cells, we also wished to examine if agonism of the receptor facilitated ROS generation in these airway epithelial cells. Using the conventional intracellular ROS generation probe CM-H_2_DCFDA, we show that stimulation of CALU3 cells with ISO (10 µM for 5 minutes) significantly increases membrane-associated ROS generation (Fig. 6A). This effect was completely abolished by pretreatment with the β-antagonist alprenolol and was approximately equivalent to that achieved by exogenous application of 1 mM H_2_O_2_ (Fig. 6A). We have previously shown that β2AR-mediated ROS generation is sensitive to NADPH oxidase inhibition^10,21^, and indeed the ISO induced DCFDA fluorescence seen here is also significantly decreased by the NOX inhibitors apocynin (APO) and diphenyleneiodonium chloride (DPI) (Fig. 6B). While ISO induces a significant level of increased ROS generation as detected by this probe, the fluorescence detected in APO and DPI treated cells was significantly less intense and was more localized to the cell membranes (Fig. 6B). Furthermore, whereas agonism with ISO caused almost all cells to bear DCFDA fluorescence, the total number of fluorescent cells was significantly reduced in cells that were pretreated with APO and DPI prior to agonism with ISO (Fig. 6C).

To examine the functional relevance of agonist-induced ROS generation in human lung epithelial cells, we aimed to determine if ISO-stimulated ROS were able to facilitate oxidation of the β2AR. Thus we evaluated the extent of dimedone labeling of β2AR upon agonism with ISO (1 μM) using the antibody-based approach shown in Fig. 1 to immunoprecipitate β2AR and probe for dimedone-labeled Cys-proteins (Fig. 6D), or alternatively, immunoprecipitate dimedone-labeled proteins and probe for β2AR (Fig. 6E) These results demonstrate that agonism with 1 μM ISO for 5 or 15 minutes significantly increased β2AR dimedone-labeling (Fig. 6D,E) and agree with our previous data demonstrating agonist-mediated feedback^21^.

## Discussion

Herein, we show for the first time that cysteine S-sulfenation of the β2AR can be modified *in situ* with dimedone and DYn-2, allowing for functional analysis of native, oxidized, and redox-deficient receptor states. In whole live cells *in situ*, our results reveal that dimedone or DYn-2 is incorporated into β2AR in a H_2_O_2_ concentration-dependent manner (Fig. 1B,D), while the effect was not seen until higher concentrations of H_2_O_2_ upon post-lysis labeling with the later probe (Fig. 1F). In all cases, there was a basal level of dimedone incorporation, suggesting some constitutive level of oxidation. Receptor oxidation consistently and robustly increased ligand binding to both transfected and endogenous β2ARs. This phenomenon occurred in both membrane and whole cell binding and was not a result of increased membrane trafficking, as it was insensitive to dynamin. The increased binding seen in oxidized receptors was saturable, fully displaceable via competition, and was specific to β2AR in that it was not observed in non-specific binding conditions. Together these data indicate that β2AR oxidation acts similar to a positive allosteric modulator of orthosteric binding, which increases ligand accessibility, albeit in this case, does not alter ligand affinity. Trapping of oxidized Cys-S-OH groups with dimedone reverted these sites, making them inaccessible in both membrane preparations and whole cells, and suggesting that the increase in ligand binding in the oxidized state is specific to cysteines existing either in the Cys-S-OH state or undergoing kinetic redox reactions that require reduction back to the thiol state, the latter of which cannot be performed in the presence of dimedone. Of note, it is entirely possible, and even likely based on our results, that there is some cysteine oxidation in the native condition, however our previous data^10,11,21^ and those shown here explicitly demonstrate that the presence of ROS (specifically H_2_O_2_) have a functional and detectable impact directly on the β2AR by increasing the number of different cysteines that are S-sulfenated or the relative number of oxidized receptors present in the cell.

Mono- and divalent cations are known to influence GPCR agonist and antagonist binding by either modulating the receptors interaction with G-proteins^39–41^, or binding directly to allosteric sites on the receptor^42,43^. Indeed, the β2AR has long-been known to be influenced by Mg^+2^ ions^44,45^, and also contains an allosteric Zn^+2^ binding site that is coordinated by His269 and Cys265 at the cytosolic face of TM6^29,46^. Both Mg^+2^ ^28,47^ and Zn^+2^ ^29,46^ increase the density of β2AR binding sites (i.e., B_max_) in a concentration-dependent manner. Similar effects describing an increase in B_max_ via allosteric activity of Mg^+2^ has been shown for other GPCRs as well^42,48^. Our data show that oxidation under the influence of H_2_O_2_ increases the B_max_ of [^3^H]-dihydroalprenolol to a similar degree of that of Mg^+2^, and that importantly, these effects are not additive and do not affect affinity, suggesting that S-sulfenation may facilitate ligand binding in a mechanistically similar fashion to the allosteric effects of Mg^+2^. Based on its link to cation coordination, it may be tempting to speculate that this effect occurs via oxidation of Cys265 to an S-sulfenic acid, however, our efforts to use ESI/MS to identify the dimedone-labeled site(s) were hampered by the inability to obtain trypsin/chymotrypsin proteolytic fragments that spanned all 13 cysteine residues, as our proteolysis only covered approximately 50% of the total protein (data not shown). Further experiments will be necessary and are underway in our laboratory to precisely localize the oxidized site(s).

Our data reveal interesting differences in the [^3^H]-dihydroalprenolol binding in clonal HEK-β2 AR versus human lung airway epithelial cells, such that oxidation increases B_max_ in both, but the effects of the dimedone trapping decreased this effect in HEK-β2AR (Fig. 2A,B) but abolished it in CALU3 membranes (Fig. 2F). Of note, a difference was also apparent in cAMP responses where oxidation increased the effect in HEK-β2AR cells (Fig. 3A), but not in CALU3 cells, however in both cell lines, the redox-deficient state had decreased cAMP responses compared to their oxidized counterparts (Fig. 3A,D). These data may suggest that the presence of greater receptor numbers in the transfected system may mask the effects of redox-deficiency (i.e., not all receptors are oxidized and dimedone-trapped), while CALU3 cells that express endogenous (and comparatively lower) levels of receptors are more broadly affected by redox-deficiency, and display more complete insufficiency in binding and function in the redox-deficient state. Additionally, the human lung epithelial cells used here may have a different, presumably higher, level of basal receptor oxidation or oxidative signals than HEK293 cells that may also contribute to the differences seen between cell lines. This hypothesis may be supported by our dimedone derivatization experiments in the absence of agonist/oxidant (Figs. 1B,E,F, and 6B,C). Nonetheless, the redox-sensitive functional changes to cAMP, CREB and ERK signaling were β2AR specific as the effects could not be reproduced by forskolin and were not a result of Gαs to Gαi switching, demonstrating β2AR-Gαs specificity. Given cysteine-S-sulfenation occurs prior to agonism in our oxidized receptor model, β2AR activation may be regulated by either the presence of a receptor S-sulfenic acid or the active reduction of a S-sulfenic acid back to the native thiol from.

While we were able to show an increase in oxidative species in CALU3 cells upon agonism with ISO (Fig. 6A-B), a limitation is that the fluorescent probe used to do so here is not specific for only ROS, as it can also be oxidized by reactive nitrogen species and one-electron oxidants. While the exact source of β2AR-mediated ROS generation is likely variable and cell-type specific, in our hands as well as described by others^12^, conventional luminol and ethidium-based probes, which are more selective for superoxide, do not yield a specific signal upon β2AR agonism, suggesting that superoxide is not the major ROS that is formed, or that perhaps its rapid dismutation to H_2_O_2_ is a more likely source. Despite its lack of absolute selectively for ROS, our results in Fig. 6 do demonstrate a clear agonist-induced oxidation of the DCF-based probe in CALU3 cells, and this effect is most pronounced along the cell membrane, where both β2AR and NOX are localized, rather than in intracellular structures such as mitochondria, which can also be sources of ROS. This can be visualized by both confocal (Fig. 6A) microscopy or using fluorescent (Fig. 6B) microscopy in the presence of the NADPH oxidase inhibitors APO and DPI, both of which show less robust total cell fluorescence, which is more peripherally restricted. The later experiments also show clear decreases in the total number of cells that fluoresce (i.e, form ROS) upon agonism (Fig. 6C) and together, these results further suggest that membrane-localized ROS-generating enzymes (e.g., NADPH oxidase), rather than mitochondria, modulate the β2AR ROS effect. Given the dearth of information in the literature on CALU3 signaling and our inability to transfect these cells, our analysis of the role of oxidation state on β-arrestin was performed in HEK-β2AR cells. β-arrestin-2 recruitment to the plasma membrane was significantly blunted in redox-deficient cells, whereas both native and oxidized receptors were able to recruit β-arrestin as normal. Furthermore, β-arrestin mediated ERK1/2 activation was abolished in the redox-deficient state, indicating the quantity of arrestin that is recruited to the redox-deficient receptors are either not activated by receptor binding or β-arrestin does not dissociate from the β2AR upon internalization as occurs with non-dimedone treated receptors^49^. Furthermore, redox-deficient receptors are unable to effectively internalize like their redox capable counterparts. Together, this data indicates redox capable cysteine residues are necessary to create an effective phosphosensor to drive canonical β-arrestin interactions.

The upregulation of NOX isoforms and resulting progressive increases in ROS generation in asthma, COPD, and other inflammatory pulmonary conditions produce a highly oxidative state^2,4^. Additionally, our laboratory as well as others, have shown that agonism of the β2AR elicits ROS generation^5,6,8–12,50,51^, and together, these data suggest that chronic use of β2AR agonists in these pulmonary diseases can lead to a significantly higher oxidative load. Here, we present the novel finding that these agonist induced ROS can in fact oxidize resident β2ARs on human lung epithelial cells, thereby completing a ROS-β2AR feedback loop. The potential physiological relevance of our findings may relate to the fact that clinically, chronic β2AR agonist use facilitates tachyphylaxis (i.e., tolerance) to the bronchodilatory effects. While receptor desensitization, internalization, and polymorphisms have been shown to play a potential role in this phenomenon^41,42^, the exact mechanisms remain elusive and the role of ROS in this effect remains completely unstudied. The major findings we report here are that receptor oxidation increases β2AR ligand binding and function, and that in every instance, redox-deficient receptors were unable to elicit the same degree of a signaling response as that seen in native or oxidized receptors. The significance of this result in the context of the heightened levels of ROS in the inflamed asthmatic or COPD-diseased lung may imply that under these conditions, β2AR is exposed to conditions that favor higher order oxidation of cysteine residues, namely Cys-S-sulfinic or Cys-S-sulfonic acids. Though we have not directly assessed the formation of these higher order species here, the dimedone-trapped model we describe herein represents an irreversible and redox-deficient state that is unable to be reduced to the Cys-S-sulfenic or Cys-S-thiolic states, resulting in a receptor state equally incapable interacting with local ROS. Additionally, given that chronic β2AR agonism would be expected to generate additional ROS and increase this oxidative potential, taken together, our data suggest that conceivably, over-oxidation of β2AR to these non-functional redox-deficient states may mechanistically account, at least in part, for β2AR tachyphylaxis seen clinically. Importantly, we also show that CREB activity is significantly impaired in the redox-deficient state, implying significant effects on downstream transcriptional outcomes. Since transcription of β2AR mRNA is itself dependent on β2AR-CREB transcriptional activity^52^, this infers that the inability of redox-deficient β2AR to modulate CREB phosphorylation would directly impact the receptors ability to regulate its own transcription. Hence, our results suggest that redox capability of resident cysteine residues are not only functionally necessary, they are also vital for stimulating transcription and downstream translation of new β2AR protein. Together, these findings may be of particular importance in the highly oxidative milieu of the asthma or COPD-diseased lung, whereby the creation of redox deficient receptors, plausibly through over-oxidation of β2AR, could facilitate functional decreases in downstream CREB-mediated β2AR expression, an effect that could also in part explain tachyphylaxis to β2-agonists. Further studies are underway in our laboratory to advance our knowledge on the role of this β2AR-ROS redox axis in normal and asthma diseased lung, and to determine whether S-sulfenic and S-sulfonic acids physiologically contribute to β2-agonist tachyphylaxis.

In summary, we report that β2AR is oxidized to Cys-S-OH *in situ*, and that receptor redox state can significantly alter β2AR ligand binding and function. These results reveal the existence of an understudied β2AR-ROS redox axis, which if perturbed, can interfere with proper receptor function.

## Supplementary information

Supplementary Information.

## Data Availability

All data generated or analyzed within this study are included in this published article and/or its Supplementary Information File.
