# Supplementary material for: Cysteine redox state regulates human β2-adrenergic receptor binding and function
Source: Sci Rep. 2020 Feb 19;10:2934. doi: 10.1038/s41598-020-59983-4 (PMC7031529; doi:10.1038/s41598-020-59983-4)
Supplement: Supplementary file 1 — Supplementary Information. [file 41598_2020_59983_MOESM1_ESM.pdf]

Cysteine redox state regulates human β2-adrenergic receptor binding and function

Kalyn M. Rambacher and Nader H. Moniri[1*]

Supplementary Information

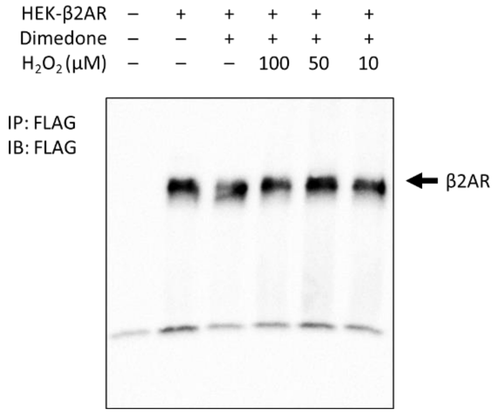

Supplementary Figure S1. Immunoprecipitation and immunoblotting with anti-FLAG antibody in untransfected cells as well as cells transfected with FLAG-β2AR.

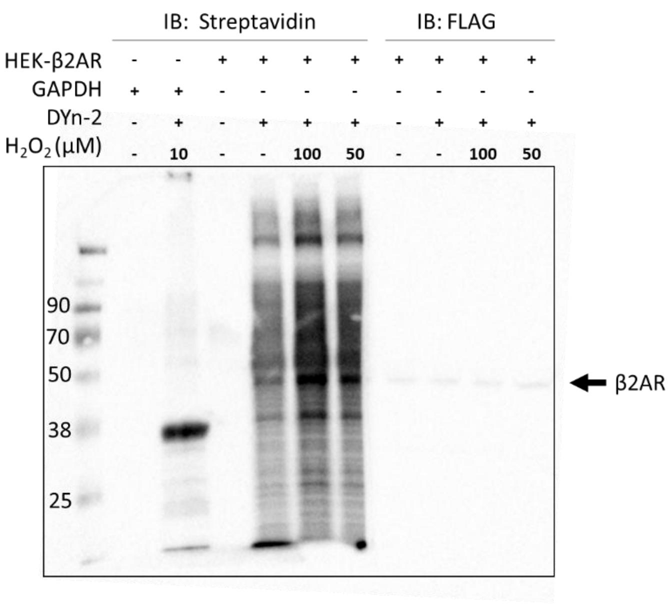

Supplementary Figure S2. Single gel, single exposure image of figure 1D.

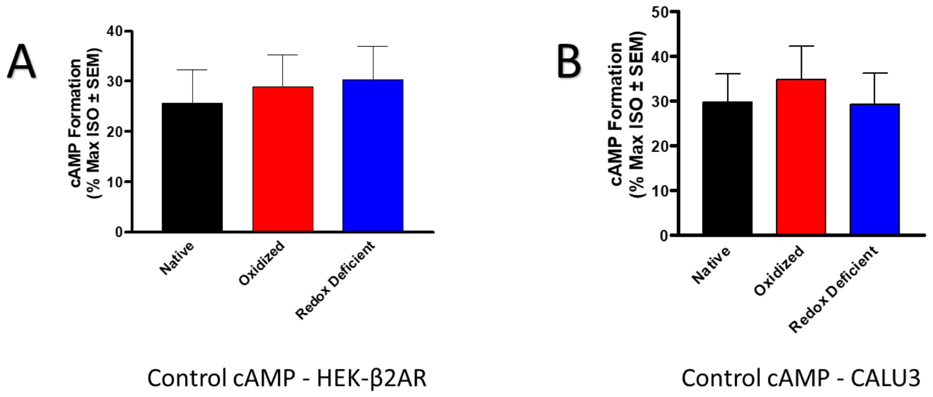

Supplementary figure 3. Control experiments demonstrate that cAMP formation in HEK-β2AR cells (A) and CALU3 cells (B) is not altered by 100 μM H_2O_2 or 1 mM dimedone alone.

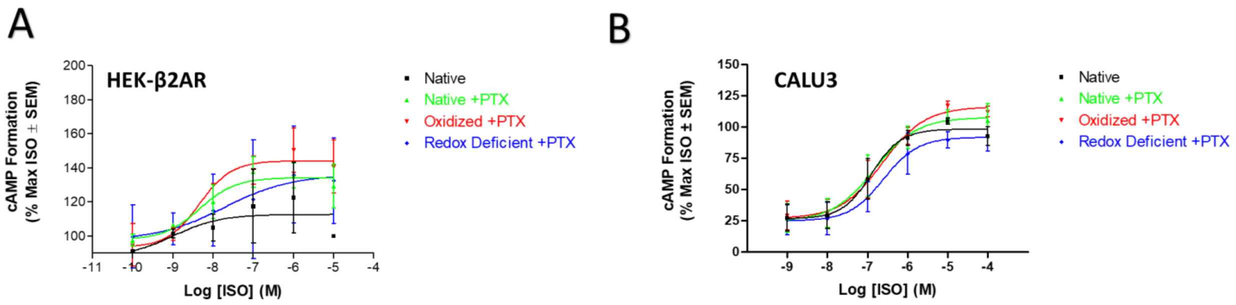

Supplementary figure 4. ISO-mediated trends in cAMP formation seen in Figure 3 were not altered by PTX in oxidized or redox-deficient HEK-β2AR cells (A) or CALU3 cells (B). (A) As in Figure 3A, oxidation still increases cAMP formation and this effect is reversed in the redox-deficient state in the presence of PTX in HEK-β2AR. (B) As seen in Figure 3D, the redox-deficient state again exhibits attenuated ISO-mediated cAMP formation compared to both native and oxidized states in CALU3 cells.

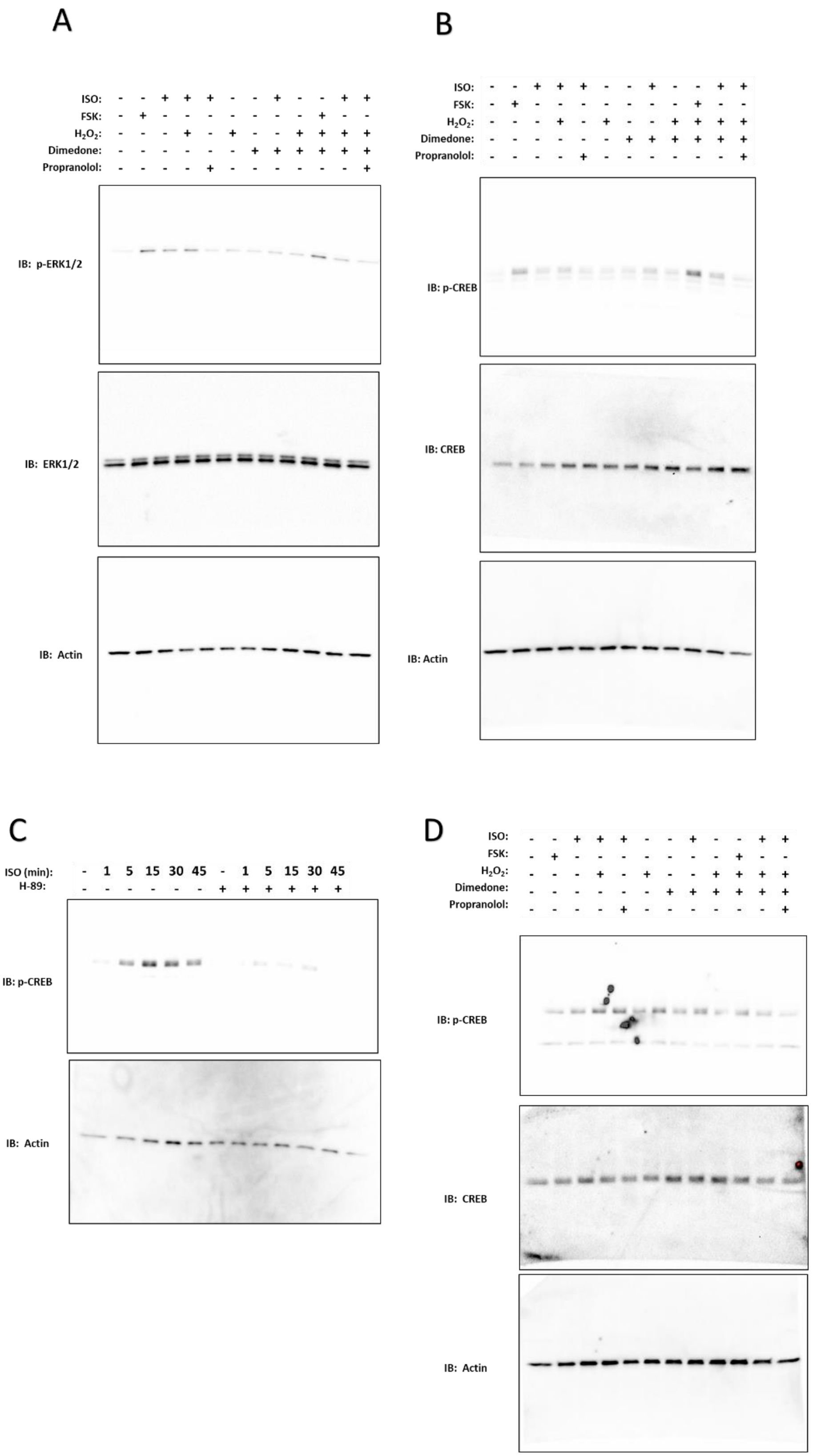

Supplementary figure 5. Full length immunoblots from figure 4.

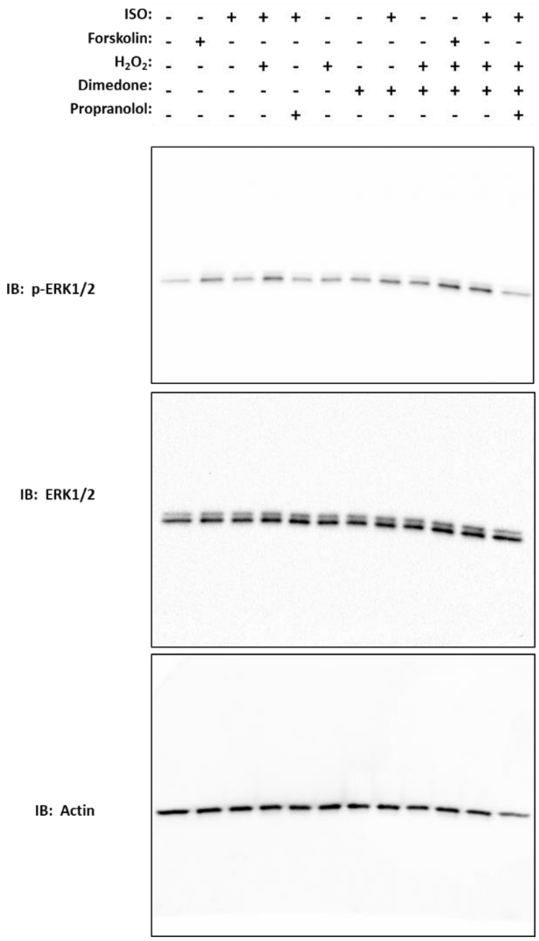

Supplementary figure 6. Full length immunoblots from figure 5B.

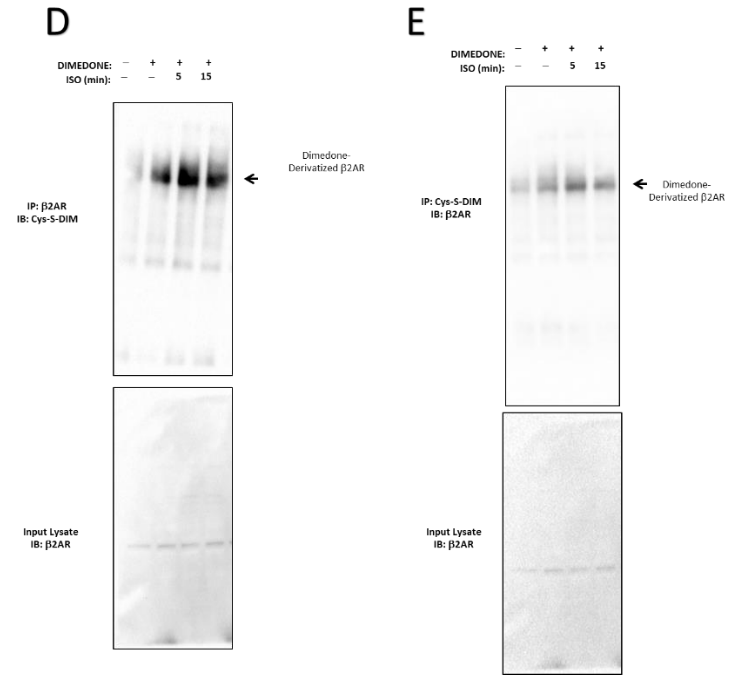

Supplementary figure 7. Full length immunoblots from figure 6D-E.
